# The largest Bio-Silica Structure on Earth: The Giant Basal Spicule from the Deep-Sea Glass Sponge *Monorhaphis chuni*

**DOI:** 10.1155/2011/540987

**Published:** 2011-09-04

**Authors:** Xiaohong Wang, Lu Gan, Klaus P. Jochum, Heinz C. Schröder, Werner E. G. Müller

**Affiliations:** ^1^National Research Center for Geoanalysis, Chinese Academy of Geological Sciences, 26 Baiwanzhuang Dajie, 100037 Beijing, China; ^2^Institute for Physiological Chemistry, University Medical Center of the Johannes Gutenberg University Mainz, Duesbergweg 6, 55128 Mainz, Germany; ^3^Biogeochemistry Department, Max Planck Institute for Chemistry, P.O. Box 3060, 55020 Mainz, Germany

## Abstract

The depth of the ocean is plentifully populated with a highly diverse fauna and flora, from where the Challenger expedition (1873–1876) treasured up a rich collection of vitreous sponges [Hexactinellida]. They have been described by Schulze and represent the phylogenetically oldest class of siliceous sponges [phylum Porifera]; they are eye-catching because of their distinct body plan, which relies on a filigree skeleton. It is constructed by an array of morphologically determined elements, the spicules. Later, during the German Deep Sea Expedition “Valdivia” (1898-1899), Schulze could describe the largest siliceous hexactinellid sponge on Earth, the up to 3 m high *Monorhaphis chuni*, which develops the equally largest bio-silica structures, the giant basal spicules (3 m × 10 mm). With such spicules as a model, basic knowledge on the morphology, formation, and development of the skeletal elements could be elaborated. Spicules are formed by a proteinaceous scaffold which mediates the formation of siliceous lamellae in which the proteins are encased. Up to eight hundred 5 to 10 *μ*m thick lamellae can be concentrically arranged around an axial canal. The silica matrix is composed of almost pure silicon and oxygen, providing it with unusual optophysical properties that are superior to those of man-made waveguides. Experiments indicated that the spicules function *in vivo* as a nonocular photoreception system. In addition, the spicules have exceptional mechanical properties, combining mechanical stability with strength and stiffness. Like demosponges the hexactinellids synthesize their silica enzymatically, via the enzyme silicatein. All these basic insights will surely contribute also to a further applied utilization and exploration of bio-silica in material/medical science.

## 1. Introduction

In the last decade, the phylogenetically oldest metazoan phylum, the Porifera (sponges) gained special interest. Mainly due to the introduction of molecular biological techniques, solid evidence was elaborated which indicated that this phylum harbors a cornucopia of new information for the understanding of the dynamics of evolutionary processes that occurred during the Earth period of Ediacara, the time prior to the Cambrian Explosion which can be dated back to approximately 540 million years ago. Furthermore, the species of this phylum are rich and valuable sources for bioprospecting, the translation of life science discoveries into practical products or processes for the benefit of the society.

Sponges are the simplest multicellular animals which are grouped to the phylum Porifera according to Grant [1]. Grant [1] described these sessile, marine animals to be built just of soft, spongy (amorphously shaped) material. Later, with the discovery of the glass sponges (class Hexactinellida) [2], this view changed drastically; they were then regarded as the “most strongly individualized, radial symmetrical” entities [3]. Since their discovery, the hexactinellids were appraised as “the most characteristic inhabitants of the great depths, which rival” with the second class of Porifera, the demosponges, “in beauty” [4]. Their thin network of living tissues is supported by the characteristic skeleton, a delicate scaffold of siliceous spicules, some of which may be fused together by secondary silica deposition to form a rigid framework [5]. The Hexactinellida together with the Demospongiae forms a common taxonomic unit comprising the siliceous sponges. Their skeletons are built of silica that is deposited in the form of amorphous opal (SiO_2_·*n*H_2_O) and constructs a variety of distinct structures termed spicules. According to molecular data from sponge genes that encode receptors and signal transduction molecules [6–8], the Hexactinellida were established to be the phylogenetically oldest class of the Porifera. Based on the discovery that the Porifera share one common ancestor, the Urmetazoa, with the other metazoans [9, 10], it was deduced that these animals represent the oldest, still extant metazoan taxon. Even more, the emergence of these animals could be calculated back to 650–665 million years ago [Ma], a date that was confirmed by fossils records [11]. Hence the Porifera must have lived already prior to the Ediacaran-Cambrian boundary, 542 Ma, and thus their elucidated genetic toolkit [8] may contribute to the understanding of the Ediacaran soft-bodied biota as well, as sketched by Pilcher [12]. It was the evolutionary novelty, the formation of a hard skeleton, that contributed significantly to the radiation of the animals in the late Proterozoic [13] and the construction of the metazoan body plan [14]. Later in evolution after the Ediacaran period [15] the third class of Porifera appeared, the Calcarea, which comprises a calcium-carbonate skeleton. 

The hexactinellid sponges are characterized by siliceous spicules that display hexactinic, triaxonic (cubic) symmetries, or morphologies derived by reduction from the basic building plans of the spicules. Their body shapes are less variable and more structured than those found in Demospongiae. The Hexactinellida have been divided into two main lineages, the Amphidiscophora and the Hexasterophora [16]. They are funnel to cup shaped and achieve the stability of their bodies by pinular pentactines, and rarely by hexactins, while the fixation to the substrate is maintained by basalia (monactines). It is the variation in the basalia that gives the Amphidiscophora their distinguished morphology. The basalia can be bundled or even balled together. The most outstanding species of this order are *Monorhaphis* and *Hyalonema* due to their sizes. The second order of hexactinellids is represented by the Hexasterophora that comprise a rigid dictyonal framework originating from simple hexactins. Their body plans typically feature branching and anastomosing forms with terminal oscular plates. The best known example is *Euplectella aspergillum*. 

The siliceous Hexactinellida and Demospongiae as well as the Calcarea, comprise spicules which apparently have the same basic construction plan. It remains enigmatic by which genetic program this complex skeleton is initiated, run and maintained. We adopt the view that the formation of the spicules, their morphology, is the primary origin of the skeleton, while the spongin cement is secondary. We hope that this paper will provide a further basis for a molecular/cell biological understanding of spicule formation in Hexactinellida, taking the giant basal spicules [GBS] from *Monorhaphis* as the model structural element since they represent the largest bio-silica structure on Earth and allow exemplarily investigations on the formation of the sponge spicules on different morphological levels. 

### 1.1. Monorhaphis chuni

**Figure 1:**
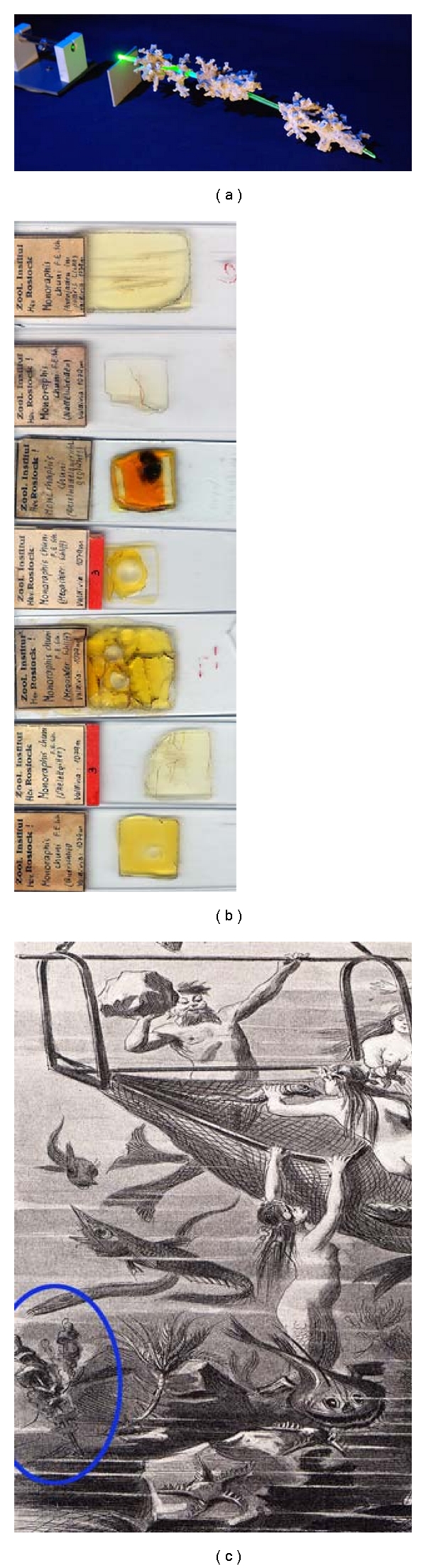
Discovery of *Monorhaphis chuni*. The hexactinellid *M*. *chuni* has first been described by Schulze [17]. (a) Original GBS which was used by Schulze for his description. (b) Glass slides prepared by Schulze for the description of the spicules. (c) Alegoric view how the scientists at that time advertised the deep sea collection of animals in general and of *M. chuni* (circled in blue) in particular to the public [18].

**Figure 2:**
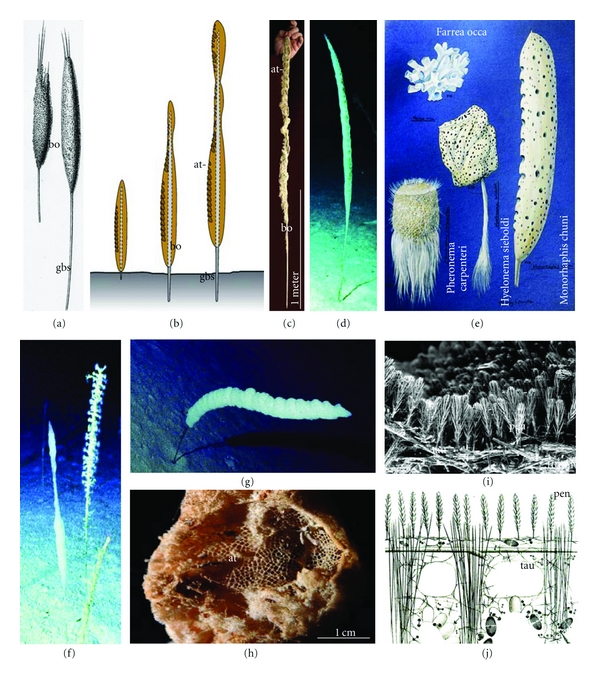
*M*. *chuni*. (a) Young specimens are anchored to the muddy substratum by one single giant basal spicule (gbs). The body (bo) surrounds the spicule as a continuous, round cylinder. (b) Schematic representation of the growth phases of the sessile animal with its GBS (gbs) which anchors it to the substratum and holds the surrounding soft body (bo). The characteristic habitus displays linearly arranged large atrial openings (at) of approximately 2 cm in diameter. With growth, the soft body dies off in the basal region and exposes the bare GBS (a to c). (c) Part of the body (bo) with its atrial openings (at). The body surface is interspersed with ingestion openings allowing a continuous water flow though canals in the interior which open into oscules that are centralized in atrial openings, the sieve-plates. (d) *M. chuni* in its natural soft bottom habitat of bathyal slopes off New Caledonia (photograph taken by Michel Roux, University of Reims; reproduced with permission). The specimens live at a depth of 800–1,000 m [23]. In this region, the sponge occurs at a population density of 1-2 individuals per m^2^. The animals reach sizes of around 1 m in length. (e) Drawing from different hexactinellids. (f and g) Living *M*. *chuni*. (h) Part of the body with one atrium (at). (i) HR-SEM image of the lattice of a grille. The pentactines (pen) are oriented towards the exterior of the body thus forming a mechanical and relative sealing of the atrial opening. (j) Grilles forming the atrial openings are composed of tauactines (tau), framing of lattices, on which the pentactines (pen) are arranged in a phalanx.

The 19th century marks the beginning of the deep sea research, when it became overt how densely populated this region of our planet is (Figure 1(c)) [19]: during the repair of a telegraph cable that was laid across the bed of the Mediterranean, was brought up in 1860 from a depth of 2000 m, and was found to be covered with mollusks, worms, and bryozoa. It then became evident that the deep sea presents a cornucopia of “exotic” species. Already Barboza du Bocage [20] described the first *Hyalonema *species (Figure 2(e)). In the following years, an armada of expeditions was sent off to explore the biotic and abiotic world of the deep sea, with the Challenger Expeditions (1870 and 1872) as the most famous and pioneering ones. The major results were published in the series “Report of the Scientific Results of the Voyage of the H.M.S. Challenger During the Years 1873–76”. One complete volume in this series was already devoted to the Hexactinellida; the material collected during this expedition was prepared and analyzed by Schulze [21]. This author was initially and primarily focused on the species * Aspergillum*, but finally also gave a first comprehensive classification of the different hexactinellids known at that time. In this compilation, Schulze [21] did not primarily concentrate on the cytological, structural, and functional aspects of the spicules but on taxonomy. However, with this opus he laid the basis for his intriguing description of the hexactinellids, with *Monorhaphis*, collected during the German Deep Sea Expedition “Valdivia” in the years 1898-1899, in the center [22]. The Chief of the Expedition Chun [17] gave in his first summary a photograph of a *Monorhaphis* specimen collected from a depth of 1644 m off the coast of East Africa (Somalia basin). This specimen had an estimated size of 3 m and surrounded one equally long siliceous spicule (Pfahlnadeln) which became one of the most lionized collected objects of that expedition (Figure 1(a)). The spicule was surrounded by stony corals (Figure 1(a)). Because of their sizes and the depths from which the specimens were collected, no complete spicule was found. Using the giant basal spicules (GBS) from this expedition, Schulze [22] provided a detailed description of their morphology and their development. His data, with their scientific accuracy, are still the reference for present day reviews.

## 2. Organism

Three species of Monorhaphididae have been described *Monorhaphis chuni* [22], *Monorhaphis dives* [22], and *Monorhaphis intermedia* [18]. These sponges (Figures 1(a) and 1(b)) are distributed in the Indo-West Pacific region and were found in depths between 516 and 1920 m [24]. *Monorhaphis* inhabits muddy substrata and is fixed there by a single GBS. Photographs from the natural environment are only available from Roux et al. (Figures 2(d), 2(f), and 2(g)) [25]. Young specimens have been imagined to comprise a continuous body, as has been sketched by Schulze [22]; one GBS anchors the specimen to the substratum and carries the cylindrical body (Figures 2(a) and 2(b)). The cylindrical/oval body of *Monorhaphis *is interspersed with many atrial openings which are located along one side (Figures 2(b), 2(c), and 2(g)). Through these openings, the regular choanosomal skeleton consisting of 14 different types of siliceous spicules can be observed (Figure 2(h)). The diameter of the body reaches in larger specimens 12 cm. During growth, the specimens elongate together with the extension of their GBSs (Figures 2(a) and 2(b)).

## 3. Spicule Diversity

**Figure 3:**
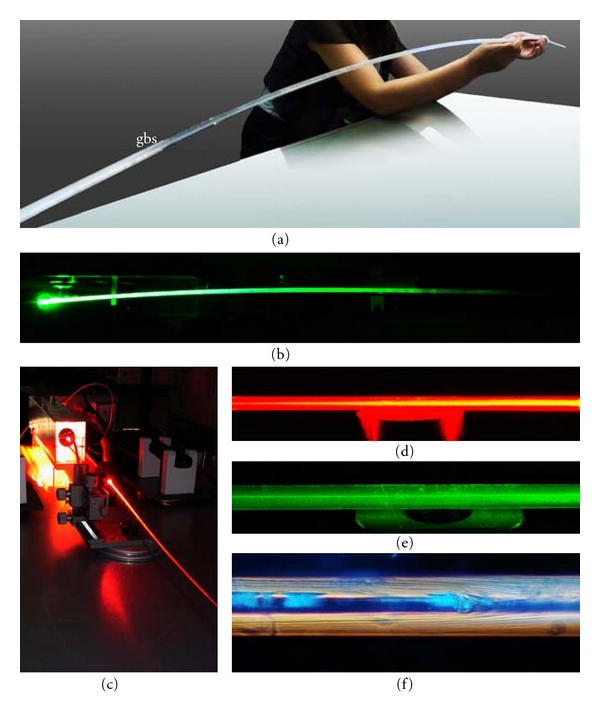
Giant basal spicules (gbs) from *M*. *chuni*. (a) Largest GBS hitherto found. GBS lighted with different laser light, green and red (b to e). The length of the spicule is 270 cm and the diameter 10 mm. (f) Illumination of a spicule with “daylight” to show the organization of the lamellae.

Like all other hexactinellids, also *Monorhaphis* possesses microscleres [<0.1 mm] (Figures 2(i) and 2(j)) as well as megascleres [0.2–30 mm to 3 m] (Figure 3(a)). Within the oblong, laterally compressed body (choanosomal body) which is arranged around the single GBS, 14 further types of siliceous spicules with lengths ranging from a few micrometers to 50 mm are found [22–24]. The likewise large comitalia (around 60 mm) support the basal characteristic habitus of this species and stabilize the tissue through which particulate food is filtrated through the aquiferous canal system of the animal. The choanosomal body comprises mainly triactines (tauactines), diactines, and amphidiscs. The hexactin spicules of the choanosome with their six nonbranched rays are arranged perpendicular to one another.

## 4. GBSs

The spicules are formed from an inorganic silica layer/mantel and an organic scaffold. The silica mantel is constructed of individual lamellae; these have been analyzed mainly by High Resolution Scanning Electron Microscopy (HR-SEM). The description here proceeds from the mm to the nm scale. 

**Figure 4:**
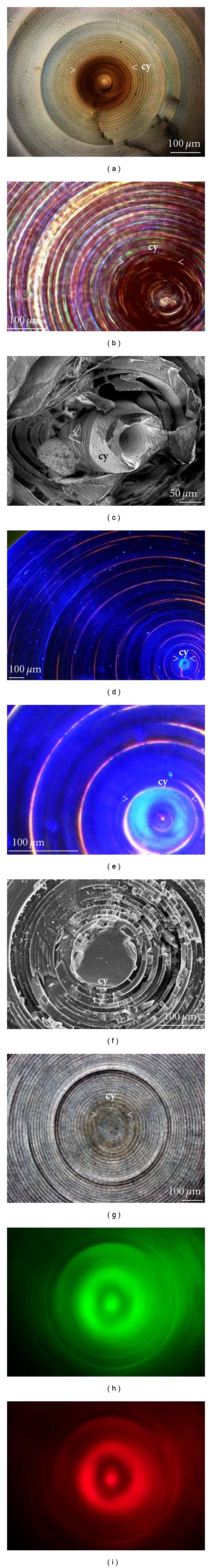
Lamellar composition of the GBS axial cylinder; light microscopic (a, b, d, e, g-i) and SEM images (c and f). The cross sections illuminated with red and green light; overlays from those images were computed. (g) A cross section illuminated with white light; the same section illuminated with green (h) or red laser light (i) to highlight that the axial cylinder is a better/more effective waveguide. The solid axial cylinder (> < cy) is marked.

A diagonal SEM analysis of a fractured comitalia (large spicules existing in the body around the atrial openings) shows already the lamellar organization of the silica mantel (Figure 4). The lamellae are arranged perfectly concentrically around the central axial cylinder (Figures 4(a) to 4(f)). If the comitalia or the GBSs are broken, the central cylinder remains almost intact, while the peripheral lamellar zone is fractured into concentric piles of chipped lamellae (Figure 4(c)).

**Millimeter Scale**

The basic microscopic architecture of the GBSs (up to 3 m long) is also identical with that of the large comitalia (~60 mm) that are found in the choanosomal skeleton of the body. The spicules are, due to their composite texture and structure, distinguished from other bio-silica structures by an unusual mechanical stability with respect to strength, flexibility, and toughness.

**Micrometer Scale**

**Figure 5:**
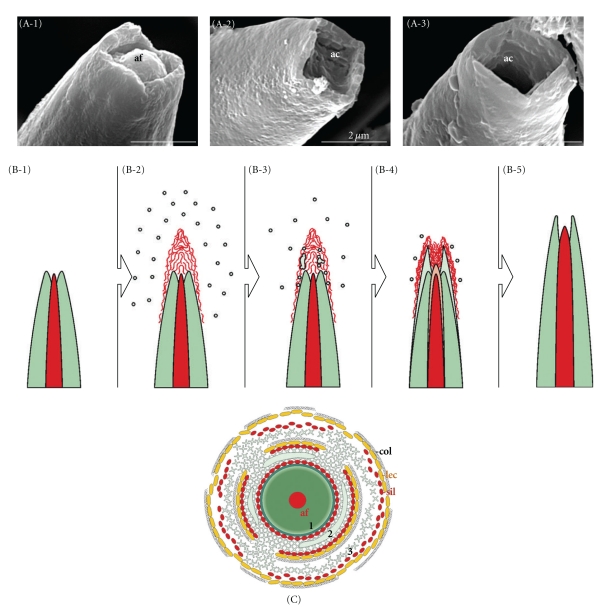
Tauactin spicules with open tips. (A-1–A-3) All spicules in Hexactinellida display a square opening of the axial canal (ac); SEM analysis. The quadrangular axial filament (af) is connected with the outer surface of the spicules and permits longitudinal growth; it also determines the direction of spicule formation. Whereas in most spicules, the opening does not contain any material (A-2, A-3), the axial canal of some spicules contains an axial filament (af; A-1). (B-1–B-5) Scheme of the longitudinal growth of the spicules. (B-1) In the initial stage, the spicule with its silica layers (dark green) has within its axial canal the axial filament (red), which reaches almost to the tip of the spicule. (B-2–B-4) During the growth of the spicule, silicatein-like material mediates the deposition of the polymeric silica (green dots and patches), which is deposited as a new layer on top of the previous silica layer (light green). (B-4-B-5). With progress of the axial growth of the spicules, the organic material becomes internalized into the spicule and contributes to the elongation of the axial filament. (C) Proposed formation of spicules in the hexactinellid *M. chuni* by appositional lamellar growth. The center of the spicule comprises an axial canal filled with an axial filament (af, red); the protein composition includes also the silicatein(-related) protein. Around the axial filament, the first lamella has been formed (1). The formation of the next silica lamella is thought to be mediated by silicatein(-related) proteins (red ellipsoid dots) arranged on both the surface of the first lamella and on a proteinaceous tube/cage stabilized in its outer layer by lectin molecules (yellow dots). The final orientation of the tube is provided by the collagen mat. Within the cage, a solid silica lamella is formed through an association of the silica clusters (left to right). During this growth process of the spicules, a thickening of the spicules takes place by the formation of new silica lamellae (2-3). The organic material of the cage undergoes proteolytic disintegration, as indicated in layer 2. The concentric arrangement of the silicatein(-related) proteins/lectin associates is proposed to be stabilized by collagen (gray fibers).

Recently published studies have been performed by HR-SEM [23, 26]. Cross sections showed a structural division of the spicules into three zones (Figure 5). In the center of the spicules lies the axial canal, which harbors the axial filament; in cross sections the axial canal has a square appearance [27–30] which is more pronounced towards the tips of the spicules. The axial canal is surrounded by a region of electron-dense homogeneous silica constituting the axial cylinder with a diameter of 100–150 *μ*m. The third and major zone of the spicules is composed of 300 to 800 regularly and concentrically arranged lamellae (each 3 to 10 *μ*m thick). The interlamellar space of the spicules is surprisingly not a continuous open slit [23]. It is in average 0.1-0.2 *μ*m wide and displays fusion zones and open spaces; apparently the fusion zones allow a continuum between two silica lamellae.

**Nanometer Scale**

Insights into the structural organization of the spicules at the nm scale can be obtained by partial and limited dissolution of the silica using hydrofluoric acid (HF) with the limitations described [31]. A rapid dissolution results in the removal of the inorganic scaffold, while gentle exposure of cross breaks of the spicules to HF vapor results in the dissolution of the silica material under release of the organic component of the lamellae [32, 33].

## 5. Chemical Composition

**Figure 6:**
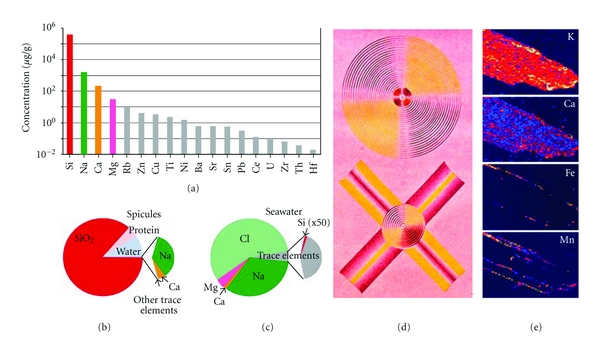
LA-ICP-MS and spectral light analyses. (a) Element concentrations (**μ**g/g) within the *Monorhaphis *spicule “Q-B”; the elements are arranged according to their abundance. Note the logarithmic scale of the abscissa. (b) Pie diagram, showing the abundance of SiO_2_, protein, and water, in comparison to the low portion of trace elements (sector part), including Na- and Ca-oxides, and further trace components. (c) A comparative diagram showing the distribution of these elements in seawater; there, Si exists as a trace element, as seen in the sector piece, whereas Cl, Na, Mg, and Ca are abundant. (d) Refraction of polarized light by spicules from the hexactinellid *M. chuni* [18]. Spectral light pattern of a cross-sectioned GBS (above) and a stauractine spicule (below) after illumination with two crossed nicol prisms. (e) Electron microprobe analysis of a GBS. The maps for the elements K, Ca, Fe, and Mn are shown.

In a first approach to understand the chemical composition of the bio-silica within the GBS, polished thin sections were prepared for electron-probe microanalysis (EPMA or electron microprobe). These analyses showed that besides of Si and O trace amounts of Ca, Fe, and Mn are present in the GBS (Figure 6). The gross chemical composition of sponge spicules has been described for both Demospongiae and Hexactinellida in general [see: [28]] and also for *Monorhaphis* in particular. Already Schulze [22] determined that, other than Si minerals (96%), only trace amounts of Na and K contribute to the inorganic material in measurable amounts (Figure 6(c)). This composition was later confirmed by Sandford [28] and Lévi et al. [34]. Based on microprobe analyses, experimental evidence has been presented indicating that Si is uniformly distributed throughout the silica shell of the spicules, whereas Na and K are not [23, 34]. Higher levels of K (around 1 wt%) have been measured in the central part of the spicules, whereas the amount dropped considerably (*≈*0.4 wt%) at the surface. The opposite is true for the distribution of Na; this level was almost negligible at the center (*≈*0.03 wt%) but increased towards the surface to *≈*0.4 wt%. However, recent studies using the Laser Ablation-Inductively Coupled Plasma-Mass Spectrometry (LA-ICP-MS; Figure 6), allowing the simultaneous determination of 40 elements at detection limits as low as ng per g and at 120 *μ*m spots, revealed an almost uniform distribution of the elements [32]. For those studies GBSs with a diameter of approximately 7 mm were systematically and completely analyzed [32]. Si was chosen as an internal standard and an SiO_2_ content of 86% (wt) was accepted [remaining: 4.6% of protein and 9% of water]. The result that the contribution of the trace elements to the total inorganic components in the spicules is less than 0.005-fold with respect to Si is of prime interest. This implies that the quality of bio-silica in the spicules is in the range of quartz grade with respect to the low concentrations of elements other than silicon and oxygen. These trace elements are split as follows: among the monovalent counterions, Na^+^ contributes to 86% (wt) [0.21% (wt) with respect to total inorganic material in the bio-silica] and among the divalent ions, Ca^2+^ to 12% (wt) [0.03% (wt)]. All other 35 remaining elements contribute with <2% (wt) only unimportantly to the inorganic composition of the trace elements in bio-silica. The impact of this finding becomes even more meaningful in comparison with the element composition of seawater. Referring to natural seawater, Na and Cl are dominant there with 32.4% and 58.5% [solid material], respectively. Mg contributes 3.9%, Ca 1.2%, and Si only 0.006%.

## 6. Mechanical Properties

From studies with *Monorhaphis*, Lévi and coworkers [34] suggested that the layered structure of the spicules has a “beneficial” effect on the mechanical properties of the spicules. Inspired by these findings, the concept of natural composite material in rigid biological systems was born and fundamentally outlined by Mayer [35]. The organic phase controls energy dissipation especially in systems that are interspersed by very thin organic layers. In continuation of this topic, Mayer et al. [36] proposed from their load-displacement studies that in *Euplectella* breakage of spicules follows a telescope-like pattern. In more recent studies we could demonstrate that the proteinaceous matrix of the *Monorhaphis* spicules (the GBSs and the comitalia) is not evenly distributed throughout the inorganic shell around the axial canal. In fact, two morphological/structural zones can be distinguished: the axial cylinder and the lamellar zone. After having described the morphology of the GBSs of *Monorhaphis*, applying modern electron microscopic techniques [23], we demonstrated that the layers setting up the lamellar zone contain one major protein (size: ~27 kDa). Based on its binding to labeled E-64, this ~27-kDa molecule could be characterized as a protease, a (silicatein-related) polypeptide.

Considering the morphological construction and the composite nature of the GBSs from *Monorhaphis*, we studied by load-displacement experiments if these properties provide them with an exceptional mechanical stability [33, 37]. The pattern of fractures within the spicules was correlated with the organization of the lamellar zone and the axial cylinder, since both areas are characterized by different bioorganic/inorganic hybrid compositions [23]. The consecutively recordable elastic responses of the spicules which are caused by cracks of distinct lamellar piles could be resolved. By this property, the spicules acquire an unusually high stability. We attribute this property, the combination of mechanical stability with strength and stiffness, to the existence of organic molecules, especially to the ~27-kDa protein existing within the inorganic rigid bio-silica material. The inner organic axial barrel stabilizing the axial cylinder is composed of rope-like filaments and provides the spicules with more mechanical flexibility and less rigidity. It must be stressed that in our studies we could not obtain conclusive results for the existence of any organic layer between the individual lamellae of the spicules. Therefore, we do not attribute the assumed viscoelastic and/or energy dissipation properties to a possible organic interphase between the lamellae but to the proteins within them.

## 7. Nanosecondary Ion Mass Spectrometry

**Figure 7:**
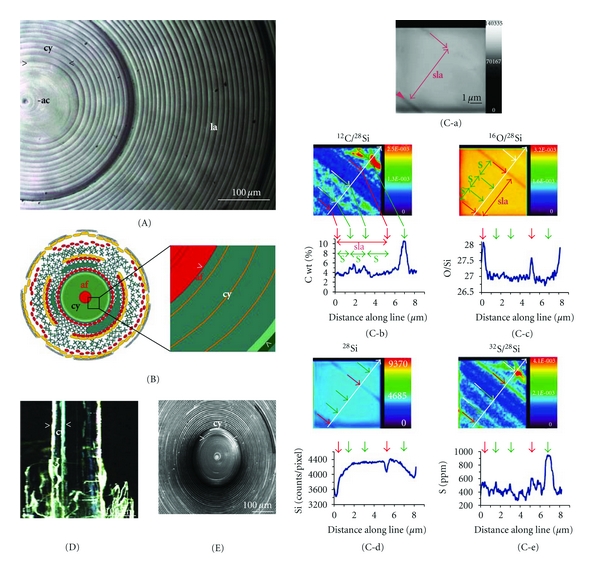
NanoSIMS images taken from a sublamella of the GBS. (A) Polished cross section through a GBS showing the three morphological regions within a spicule, the central axial canal (ac), the surrounding axial cylinder (cy), and the lamellar region (la). Light microscopic image. (B) Schematic illustration of the cross section through the GBS outlining the growth of the spicules with emphasis on the outlining the axial cylinder (cy) of which lamellae have been “biosintered”. The measurements have been performed in the boxed area. The axial cylinder surrounds the axial filament (af). (C) NanoSIMS analyses. (C-a) Image taken simultaneously with the NanoSIMS by secondary electrons; the sublamella is marked (sla) and has a thickness of 5 *μ*m. (C-b to C-e) NanoSIMS mapping to determine the distributions of ^12^C, ^28^Si, ^18^O, and ^32^S. (C-b) The pseudocolor image reflects the changes of the ^12^C/^28^Si-ratio along the indicated line field. Below: the ^12^C concentrations are calculated based on the ^12^C/^28^Si-ratio and applying the relative sensitivity factors. (C-c) ^16^O/^28^Si-ratio shows the homogeneity of the “biological glass” within the lamellae. The absolute ratio is caused by the difference of the ionization probability of silicon and oxygen in this matrix. Mapping of (C-d) silicon; the total counts of ^28^Si are given, and of (C-e) sulfur and silicon; the ^32^S/^28^Si ratios are computed. Either (absolute) concentrations or the ratio of concentrations are given as pseudocolor images. Different colors correspond to different intensities of signal or ratio, increasing from black to red. Below the color images the corresponding line-scan data are given. In (C-a), (C-b), and (C-c), the hierarchical composition of one 5 *μ*m sublamella (sla; double-headed arrow; bordered by red arrows) from three slats (s; framed by green arrows), displaying widths of about 1.5 to 1.8 mm each, is indicated. (D) and (E) Light and SEM microscopical images showing the location of the axial cylinder (cy).

**Figure 8:**
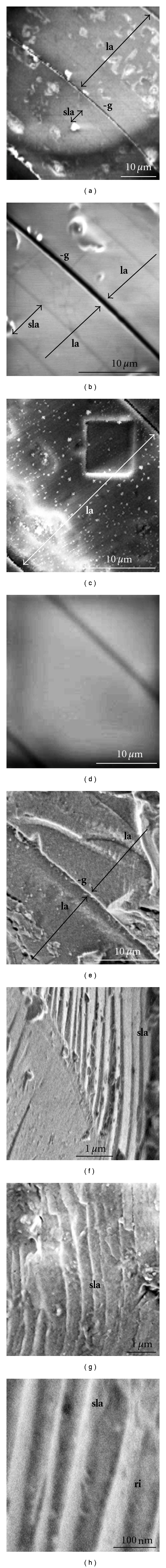
Presence of the three sublamellae within the lamella where the NanoSIMS analysis has been performed. (a) to (e) The 18 *μ*m thick lamella (la) shows a substructuring into three sublamellae (sla). In contrast to the lamellae, the sublamellae are not delimited by visible gaps (g). At higher magnifications, the spacing of the gap (g), separating individual lamellae (la), and the absence of any gap/slit between the sublamellae (sla) is more distinct. (e) The location of the area ([quadratic area]) within a lamella that had been analyzed by NanoSIMS is shown. (f) to (h) View of a GBS cross-fracture, obtained by mechanical breaking, showing a ribbed surface. Magnification at the submicron level reveals that the surface of the fracture is a rib-like corrugated sheet (ri) without gap.

Nanosecondary ion mass spectrometry (NanoSIMS) has been performed to obtain a further insight into the silica material [38]. The selected GBS used for NanoSIMS comprised 243 concentrically arranged lamellae (Figure 7(a)) with an axial cylinder of 250 *μ*m. The lamellae nearest to the axial cylinder are thicker (10–30 *μ*m) than those which exist more distantly, towards the surface of the spicules (2–10 *μ*m; Figure 8). The larger lamellae (10–30 *μ*m), surrounding the axial cylinder, were analyzed by HR-SEM and found to be separated from each other by 50 and 100 nm wide gaps (Figures 8(a) to 8(e)). Closer inspection by NanoSIMS revealed that those lamellae are composed of substructures that are not delimited by gaps but are closely packed; these were termed sublamellae. Hence, every lamella is formed of three to six stacked solid sublamellae, each measuring 3–6 *μ*m (Figures 8(f) to 8(h)). 

The distribution of C, O, S, and Si has been investigated on a 7 × 7 *μ*m^2^ area, spanning a total of three sublamellae with one complete sublamella in the middle (Figure 7(C-a)). The NanoSIMS 50 ion microprobe, operating in the multicollection detector mode, allowed a simultaneous imaging of C^−^, O^−^, Si^−^, and S^−^. C, S, and O were normalized to Si in order to minimize crater effects, which occur towards the edge of the image (Figure 7(C)). Normalization of the signals to silicon (^28^Si) revealed that the peak signals obtained followed distinct lines which correspond to the borders of the sublamellae (Figure 7(C-c) and 7(C-d)). The borders of the sublamellae are especially highlighted in the scan obtained from the ^16^O^−^/^28^Si^−^ mapping. The ratio of C and Si (Figure 7(C-b)) and the ratios of these two elements [^12^C^−^/^28^Si^−^] indicate a further substructure within the sublamella, [5 *μ*m thick] the 1.6–1.8 *μ*m subsublamellae, which we term here cylindrical slats. It is obvious that this particular sublamella, from which the mappings were derived, is composed of three slats (Figure 7(C-c)). The ratio ^12^C/^28^Si indicates that the C concentrations within the slats differ from each other (Figure 7(C-b)). It is interesting to note that the highest ^12^C/^28^Si values were not found at the borders of the sublamella but between two slats. The determination of the relative level of Si, given in counts/pixel, revealed the highest level within the sublamellae and showed a distinct decrease at their “borders” (Figure 7(C-d)). Finally, the ^32^S^−^/^28^Si^−^ ratios were determined along the same lines (Figure 7(C-e)). Based on these mapping data, we can conclude that the lamellae are hierarchically built from three to five sublamellae which are composed of three slats each. This new insight into the highly ordered distribution of protein within lamellae and lamellar substructures confirms earlier findings, revealing that the silica nanospheres built from 2.8 nm small colloids and reaching sizes of 50–200 nm in diameter are arranged within the GBS in a highly ordered, concentric manner.

## 8. Optophysical Properties

Sponges can react fast to physical stimulation from the environment with contraction or expansion. Morphological and cellular structures for such responses are conceivable based on the complex cell-cell- and the diverse cell-matrix-interaction systems in sponges that have already been detected [see: [8]]. These observations could imply that the coordinate reactions are governed by a nerve system. However, until now no nerve fibers or synapses could be identified in sponges. Nevertheless, our previous studies showed that the siliceous demosponges *Suberites domuncula *and *Geodia cydonium *contain and express genes coding for neuronal molecules, for example, a metabotropic glutamate/GABA [*γ*-aminobutyric acid-] like receptor [39]. 

Especially suitable for optophysical studies are the longer *Monorhaphis* GBSs, as well as the stalk spicules of *Hyalonema sieboldi *[40]. Using those spicules, comparable and more extensive studies have been published [26, 40]. The giant spicules (*Monorhaphis*) were exposed at their end with the wider diameter to a white light source with a spectrum ranging from 400 nm to >1600 nm (Figures 3(b) to 3(f)). Even with the naked eye, the optical waveguide properties of the GBS could be recorded. During the passage of the light, a distinct white-to-red color gradient along the spicules of glass sponges was seen, suggesting a (partial) scattering of the light (Figure 3(c)). In a closer view, it became obvious that the guided light in the paraxial region of the spicule has a bright yellow color in contrast to the light quality at the outer surface. The spectrum of the output of the light source was measured between 400 nm and >1600 nm. A distinct cut-off of the wavelengths below 600 nm and above 1400 nm could be measured, while the light transmission between these borders was only slightly/gradually reduced. Hence, the spicules act as optical fibers [like a high pass filter] cutting off the light of wavelengths below about 600 nm from transmission by more than 2 orders. A similar cut-off of the spicule was observed in the infrared wavelength range above 1400 nm. Here light transmission was blocked like a low pass filter. Three weaker absorption minima were observed on the overall profile at the centre wavelengths at around ~960 nm and ~1150 nm which were attributed to the existing water. Within the visible part of the spectrum transmitted by the fibers, less pronounced absorption wavelength regions were found which matched the molecular absorption lines of water (970 nm and 1150/1190 nm, resp.) [41].

**Figure 9:**
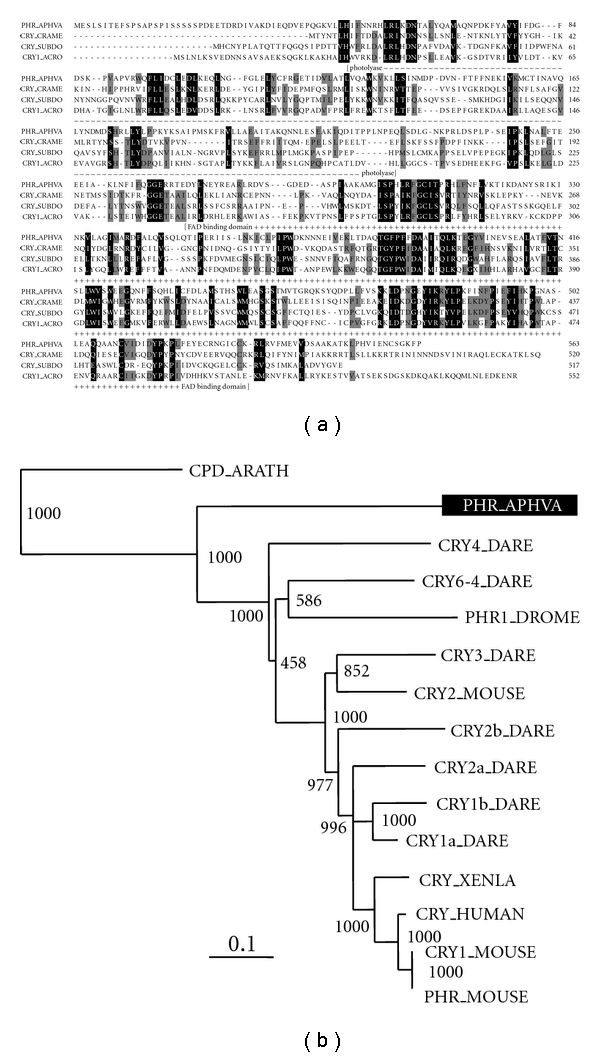
Poriferan cryptochromes. (a) The deduced poriferan cryptochrome protein sequences CRY_SUBDO (*Suberites domuncula* (CRYPTO_SUBDO; accession number FN421335), CRY_CRAME (*Crateromorpha meyeri*; FN421336), and the photolyase-related protein from *Aphrocallistes vastus* (PHR_APHVA; AJ437143.1) were aligned with the coral (*Acropora millepora*) cryptochrome CRY1 (CRY1_ACRO; 145881069). Residues conserved (identical or similar with respect to their physicochemical properties) in all sequences are shown in white on black; those which share similarity in three sequences are shown in black on gray. The characteristic domains the N-terminal photolyase-related region (photolyase), and the FAD-binding domain, are marked. (b) Phylogenetic relationship of the photolyase/cryptochrome polypeptides. The sponge photolyase-related molecule (PHR_APHVA) has been aligned with related sequences; finally a rooted tree has been computed. The numbers at the nodes are an indication of the level of confidence for the branches as determined by bootstrap analysis (1000 bootstrap replicates). The scale bar indicates an evolutionary distance of 0.1 aa substitutions per position in the sequence. The following sequences have been included [“class I photolyases”]. The cryptochrome sequences from *Danio rerio* zcry1a (CRY1a_DARE; AB042248/AB042248), zcry1b (CRY1b_DARE; AB042249/AB042249.1), zcry2a (CRY2a_DARE; AB042250/AB042250.1), zcry2b (CRY2b_DARE; AB042251/AB042251.1), zcry3 (CRY3_DARE; AB042252/AB042252.1), zcry4 (CRY4_DARE; AB042253/AB042253.1), and (6–4) photolyase (CRY6-4_DARE; AB042254), the human photolyase (CRY_HUMAN; D83702), mouse photolyase/blue-light receptor homolog 1 (CRY1_MOUSE; AB000777) and homolog 2 (CRY2_MOUSE; AB003433), frog cryptochrome 1 (CRY_XENLA; AY049033), the photolyase from *D. melanogaster *(PHR1_DROME; BAA12067.1). The *Arabidopsis thaliana* class II photolyases (CPD photolyase; CPD_ARATH; CAA67683.1) were used as outgroup to root the tree.

In a first approach, a cryptochrome [CRY] sequence from the hexactinellid sponge *Aphrocallistes vastus*, that comprises high sequence similarity to genes encoding (6–4) photolyases and related proteins, has already been identified [26]. Earlier, functional studies showed that this gene codes in *S. domuncula* for a photolyase-related protein [42]. Based on sequence similarities, the DNA photolyase from *A. vastus* has been classified together with the cryptochromes, which include blue-light receptors, into a single DNA photolyase/chryptochrome protein family (Figure 9) [26]. Taking this experimental finding together with the demonstration of the luciferase in *S. domuncula*, we propose that sponges are provided with an unusual, (perhaps) unrecognized photoreception system. We postulate that sponges coordinate their sensory reception systems not through a protein-based nervous network alone, but primarily through a siliceous spicular meshwork and nerve-cell-related sensory molecules at the ends of those spicules. Since sponges are provided with the genetic machinery to express luciferase enzymes and also a photolyase/chryptochrome molecule, the optical fibers [spicules] might guide and convert the light, via a chemical/photoelectric reaction, into electric signals. The subsequent amplification system which translates the electric signals into the nervous transmission system in sponges as well as to other metazoan phyla might be mediated by similar biological amplifiers/receptors.

## 9. Synthesis of GBS

As outlined earlier [43, 44] the initial phase of spicule formation proceeds intracellularly in sclerocytes, where the spicules elongate up to 8 *μ*m. These cells are loosely embedded in the mesohyl and usually start to synthesize several spicules simultaneously; the lengths of the spicules observed reach values of 0.7–8 *μ*m and diameters of up to 0.9 *μ*m. 

Silicatein, the major structural protein in the GBS and also the enzyme that mediates the synthesis of polymeric silica, is present not only in the axial canal, but also in the extra-spicular and extra-cellular space [43, 45]. Recently Ehrlich et al. [46] got some experimental evidence that spicules in hexactinellids contain collagen onto which they deposit silica.

**The Intracellular Phase of Spicule Formation in Sclerocytes**

Silica is actively taken up by a Na^+^/HCO_3_^−^[Si(OH)_4_] cotransporter [47]. In the first steps silicatein is synthesized as a proenzyme (signal peptide-propeptide-mature enzyme: 36.3kDa) and processed via the 34.7kDa form (propeptide-mature enzyme) to the 23/25kDa mature enzyme. Very likely during the transport through the endoplasmic reticulum and the Golgi complex, silicatein undergoes phosphorylation and is transported into vesicles where silicatein forms rods, the axial filaments. After assembly to filaments, the first layer(s) of silica is (are) formed. Finally the spicules are released into the extracellular space where they grow in length and diameter by appositional growth. The immature spicules are extruded from the pinacocytes.

**Extracellular Phase (Appositional Growth)**

Silicatein is present also in the extracellular space. As mentioned, the immunogold electron microscopic analysis showed that the silicatein molecules are arranged along strings, which are organized in parallel to the surfaces of the spicules. In the presence of Ca^2+^, silicatein associates with galectin and allows the appositional growth of the spicules. Since the surface of a newly siliceous spicule is also covered with silicatein, the appositional growth/thickening of a spicule hence proceeds from two directions [axial (Figure 5(B)) and radial (Figure 5(C))].

**Extracellular Phase (Shaping)**

In the next step, the galectin-containing strings are organized by collagen fibers to net-like structures [45]. It is very likely that collagen, which is released by the specialized cells the collencytes, provides the organized platform for the morphogenesis of the spicules. The longitudinal growth of the spicules can be explained by the assumption that at the tips of the spicules, the galectin/silicatein complexes are incorporated into deposited bio-silica under formation and elongation of the axial canal.

## 10. Concluding Remarks

Until 15 years ago, the Porifera [sponges] were an enigmatic animal taxon whose evolutionary origin, its phylogenetic position, and its genetic toolkit were largely unknown. The discovery of one protein, the cell adhesion molecule galectin, clarified those questions almost suddenly [48]. Cloning and functional studies of that molecule solved the question on the evolutionary origin of the multicellular animals by the demonstration that all metazoan phyla including the Porifera originate from one common ancestor, the hypothetical Urmetazoa [48]. After having established the monophyly of animals and having underscored the relevance of the phylum Porifera for the elucidation of the deep phylogeny of animals [9], it could be resolved that among the three classes in the phylum Porifera the evolutionary oldest class is represented by the Hexactinellida [7]. This insight came as a surprise, since the members display the most sophisticated body plan among the sponges. This enlightenment was supported and flanked by the realization that sponges comprise (almost all) basic functional circuits known also from higher metazoan phyla. Regardless of that progress, one main issue remained mysterious, the genetic basis for the construction of the highly complex skeleton built of spicules. Focusing on the siliceous sponges, major progress has been made in the understanding of the formation and the development of the spicules in the last few years. Furthermore, with the availability of the GBSs from *Monorhaphis*, substantial advances in the insight of the construction of the siliceous spicules have been achieved as outlined in this paper.
